# Epidemiologically-based strategies for the detection of emerging plant pathogens

**DOI:** 10.1038/s41598-022-13553-y

**Published:** 2022-06-29

**Authors:** Alexander J. Mastin, Frank van den Bosch, Yoann Bourhis, Stephen Parnell

**Affiliations:** 1 Ecosystems and Environment Research Centre, School of Science, Engineering and Environment, University of Salford, Greater Manchester, M5 4WT UK; 2 Department of Biointeraction and Crop Protection, Rothamsted Research, Harpenden, Hertfordshire AL5 2JQ UK; 3 Visiting Scholar, Quantitative Biology and Epidemiology Group, Plant Pathology Department, University of California, Davis, One Shields Ave, Davis, CA 95616 USA; 4 Present Address: Epidemiology and Risk Policy Advice, Veterinary Advice Services, Animal and Plant Health Agency, Nobel House, 17 Smith Square, SW1P 3JR, London, UK; 5 Present Address: Warwick Crop Centre, School of Life Sciences, University of Warwick, Wellesbourne Campus, Warwick, CV35 9EF UK

**Keywords:** Ecological modelling, Plant ecology, Entomology

## Abstract

Emerging pests and pathogens of plants are a major threat to natural and managed ecosystems worldwide. Whilst it is well accepted that surveillance activities are key to both the early detection of new incursions and the ability to identify pest-free areas, the performance of these activities must be evaluated to ensure they are fit for purpose. This requires consideration of the number of potential hosts inspected or tested as well as the epidemiology of the pathogen and the detection method used. In the case of plant pathogens, one particular concern is whether the visual inspection of plant hosts for signs of disease is able to detect the presence of these pathogens at low prevalences, given that it takes time for these symptoms to develop. One such pathogen is the ST53 strain of the vector-borne bacterial pathogen *Xylella fastidiosa* in olive hosts, which was first identified in southern Italy in 2013. Additionally, *X. fastidiosa* ST53 in olive has a rapid rate of spread, which could also have important implications for surveillance. In the current study, we evaluate how well visual surveillance would be expected to perform for this pathogen and investigate whether molecular testing of either tree hosts or insect vectors offer feasible alternatives. Our results identify the main constraints to each of these strategies and can be used to inform and improve both current and future surveillance activities.

## Introduction

Increases in international travel, transportation, and trade have increased the risk of introduction of plant pests and pathogens into new areas, with changes in land use and climate potentially facilitating their establishment and spread^1–3^. Surveillance activities in presumed “pest free areas”^4^ are required to either confidently declare pest or pathogen absence (in order to facilitate trade activities) or to detect new incursions at a sufficiently early stage for control measures to be applied^5^, and are thus commonly referred to as “detection surveys”. To date, detection surveys are generally based upon the visual inspection of economically or ecologically important host species by trained surveyors^4^. Whilst this strategy is invaluable for the detection of novel and unexpected pests and pathogens, there are concerns that it may be less effective in cases where the pest or pathogen is known but symptoms do not immediately become apparent. This is evidenced by the fact that many emerging pests and pathogens are first detected at a point in epidemic development at which control is no longer feasible^6–9^. Our own previous work on plant pathogens has demonstrated that, along with the epidemiology of the pathogen, the detection method has an effect on the number of hosts which must be inspected or tested for detection surveys to be effective^10–15^. Although new diagnostic methods capable of detection of infection in presymptomatically infected hosts^16^ offer great potential for improving detection in individual hosts, less is known about their value for large scale detection surveys—particularly as they will generally cost more than visual inspection to deploy^17^. In some cases, there is also the question of whether hosts should be tested at all. Many plant viruses and some notable bacterial plant pathogens are spread by insect vectors, which may themselves be valuable alternative sources of surveillance data^13^, yet are generally only currently used as an adjunct to conventional host-based surveillance.

The challenges facing visual inspection as a surveillance strategy are exemplified by the recent emergence of a novel strain of the vector-borne plant pathogenic bacterium *Xylella fastidiosa* in Europe. This strain (*X. fastidiosa* subspecies pauca, ST53—hereafter *X. fastidiosa* ST53) was identified in 2013 as the cause of a novel disease of olive trees (olive quick decline syndrome; OQDS) in the Italian province of Lecce in the region of Apulia^18,19^. Following first identification, the meadow spittlebug, *Philaenus spumarius*, was identified as the most important vector of this pathogen^20–23^ and the limits of infection within the Salento peninsula were identified through a delimiting survey. Whilst elimination of infection from this area is considered unlikely, *X. fastidiosa* is considered one of the greatest phytosanitary threats in Europe, meaning that there is now a need for effective surveillance in areas considered still free of infection^24^. Although much of this surveillance to date has been based upon visual inspection, the long “presymptomatic period” before hosts become visibly detectable and the high potential spread rates of *X. fastidiosa* raise questions of the efficacy of this strategy^25^. By building upon our earlier work^11–13,15^, we consider here whether visual detection can continue to be justified as the standard surveillance strategy prior to *X. fastidiosa* incursion, in the face of alternatives such as molecular testing of either hosts or vectors by considering the following questions:

- Is visual inspection useful for detection surveys?
- What characteristics of a host diagnostic test would make it more cost effective than visual inspection?
- Could laboratory testing of vectors outperform visual inspection?

## Methods

### Is visual inspection useful for detection surveys?

A single detection survey can result in one of two potential outcomes:

i. At least one positive detection is made, usually after a series of monitoring rounds where no detections are made. Assuming that the detection method in use has a perfect specificity—that is, there are no ‘false positive’ results—this indicates that the pathogen is definitely present in the population.
ii. No positive detections are made, in which case the pathogen may or may not be present in the population, due to imperfect test sensitivity (i.e. ‘false negative’ results) and/or random error (i.e. the possibility that infected hosts are present but were not sampled).

Whether or not the pathogen of interest is found during this detection survey, we are interested in answering the same general question: “given these results, what can we say about the prevalence of infection in this area?”. If a pathogen is not detected, we commonly reformulate this question in relation to a predefined “prevalence threshold” and ask what the probability is that the prevalence is lower than this threshold. If this probability is sufficiently high, it can be interpreted as evidence that the pathogen is effectively absent from the region in question^24,26^. This interpretation links well with our previous work on pest freedom determination^11^, which allows us to estimate the probability density, $P\left(q\right)$, of the prevalence, $q$, for any given sampling rate and thus identify the prevalence above which only a small percentage of the probability density remains. By changing the number of hosts inspected (and found to be negative), we can estimate the number of hosts which would need to be sampled in order for this prevalence to be lower than a given prevalence threshold, and therefore confidently declare pest freedom. This interpretation differs from conventional prevalence estimation, in which a point estimate and an uncertainty range is provided. In pest freedom surveys, we effectively only consider an upper limit of the uncertainty range and do not attempt to estimate what the true prevalence is (indeed, it is hoped that the true prevalence is 0). For ease of calculation, we consider a single detection survey, allowing us to disregard the interval between survey rounds^11^. However, our methods are also applicable to multiple rounds of a detection survey across years (either to determine pest freedom or in the case of early detection), as detailed in Supplementary Information A.

**Figure 1:**
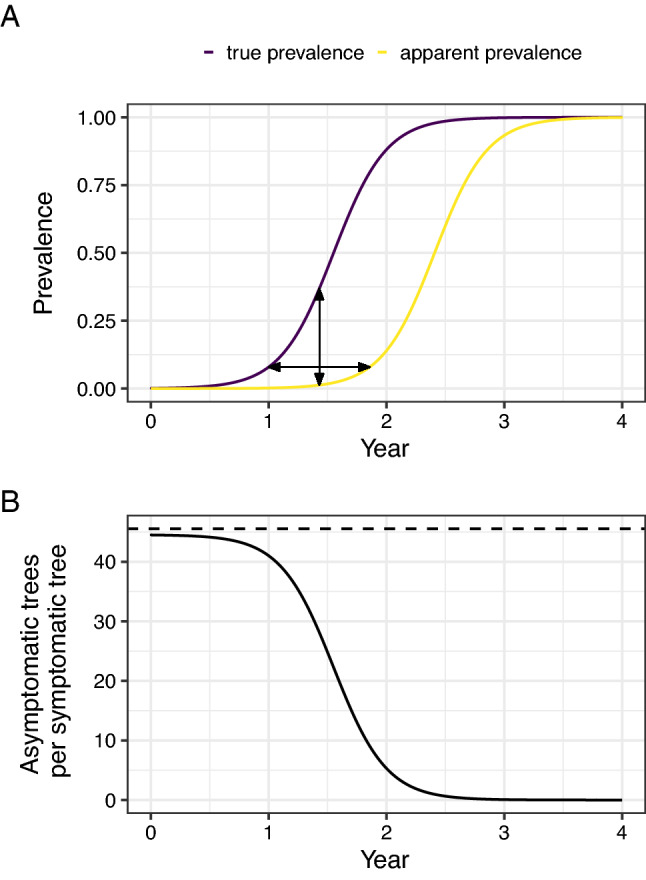
The true prevalence of infection can be estimated for any apparent prevalence from the rate of pathogen spread and the length of the detection lag period. (**A**) A detection lag period can be considered as a shift of the epidemic growth curve to the right. In this plot, time is shown on the x-axis and the proportion of infected or symptomatic hosts (the prevalence) on the y-axis. The two curves represent the true prevalence and the apparent prevalence (e.g. the proportion of hosts with symptoms, if detection is based upon visual inspection). The curves are parameterised based upon *X. fastidiosa*, but are intended for visualising the relative difference in true and apparent prevalences rather than the exact prevalences at different time points. The horizontal distance between the curves (i.e. in the direction of the x-axis) represents the asymptomatic period (the detection lag $\left(\delta \right)$ for visual inspection), and the vertical distance (in the direction of the y-axis) represents the difference between the true and apparent prevalences at any given time. (**B**) The ratio of the true and apparent prevalences decreases as the true prevalence increases. This plot shows the ratio of the true and apparent (detectable) prevalences (which can be interpreted as the number of asymptomatic trees per symptomatic tree) under logistic and exponential growth as time progresses. The dashed line represents the predicted ratio under continued exponential growth and the solid line represents that under logistic growth. Although during very early stage spread, the growth in both the true and apparent prevalences is broadly exponential, as the true prevalence deviates from this, the ratio of the two prevalences starts to decrease.

When our detection survey is based upon visual inspection, our ability to detect infection will depend upon the proportion of symptomatic hosts at any given time, which we term the “apparent prevalence”. However, we wish to declare pest freedom in relation to the true prevalence (the proportion of infected hosts, symptomatic or not). The relationship between the apparent and true prevalences will be affected by both the duration of the presymptomatic period (which we term the “detection lag”) and the rate of pathogen spread $\left(r\right)$ (Fig. 1). The ratio of true and apparent prevalences (which describes the number of infected hosts for each symptomatic host) would also be expected to reduce over time as density dependent constraints reduce the rate of increase in the true prevalence, until the true and apparent prevalences are equal (Fig. 1B). As this effect will be most pronounced when a pathogen is spreading rapidly and the detection lag is relatively long (as is the case with *X. fastidiosa* ST53), we explicitly consider this logistic growth pattern in our model^27^, rather than the exponential approximation (i.e. an assumed fixed ratio of true and apparent prevalences over time) we have previously described^10,11^. Using this approach, we are able to estimate the number of trees that would need to be visually inspected to detect a maximum true prevalence of 0.01 at a confidence level of 0.90 (Supplementary Information A), for a range of different tree pathogens—including *X. fastidiosa*.

### What characteristics of a host diagnostic test would make it more cost effective than visual inspection?

Can we improve upon the performance of a detection survey by using a laboratory test capable of identifying infection in presymptomatic hosts? The methods described above allow us to explore the impact of varying different test characteristics (namely the detection lag period and diagnostic sensitivity), but we also need to consider how the costs of alternative detection methods compare to those of visual inspection. To do this, we adapt our previous work on early detection surveillance (in which the pathogen is detected)^12^ to the situation in which there is no detection (Supplementary Information A). Rather than specifying a particular detection method, we investigate what combinations of detection lag and diagnostic sensitivity and relative cost would be required to outperform visual inspection, assuming a single round of sampling. However, we also consider the specific example of a molecular diagnostic which costs €14.63/host to deploy, in contrast to visual inspection at €5.48/host, based on estimates of the costs of *X. fastidiosa* surveillance in Apulia (Supplementary Table 2).

### Could laboratory testing of vectors outperform visual inspection?

Vector-borne pathogens such as *X. fastidiosa* can be detected in insect vectors as well as in the plant host, and our previous work has shown that the relative prevalences in vectors and hosts during early stage spread is a key consideration when identifying the value of surveillance in either group^13^. However, not only is very little known about how the prevalence of *X. fastidiosa* in vectors and hosts relate to each other during early stage spread, there is also marked seasonality in vector infection which makes capturing these relative prevalences more challenging. *P. spumarius* is univoltine (i.e. a single new generation is produced per year) and adults rarely survive the winter months. The total density of adult vectors therefore rapidly increases from the time of first emergence in spring, to peak in summer, before decreasing to very low levels over the winter months due to mortality^28^. As *X. fastidiosa* is lost during moulting and is not transmitted vertically, adult vectors (which are motile and therefore the main source of tree to tree spread^29^) would be expected to only acquire infection in a relatively short window following emergence in spring and whilst feeding on potentially infected olive hosts (before moving to herbage in late summer). In Apulia, the prevalence of vector infection therefore rapidly increases in the Spring and Summer months, before reducing to very low levels over winter each year as remaining adults die off^20,22,28^. Finally, because *X. fastidiosa* is a ‘semipersistent’ pathogen^30,31^, it is restricted to the foregut of infected vectors, meaning that colonised tissue can be more reliably isolated at an early stage in infected insect vectors than in infected plant hosts.

**Figure 2:**
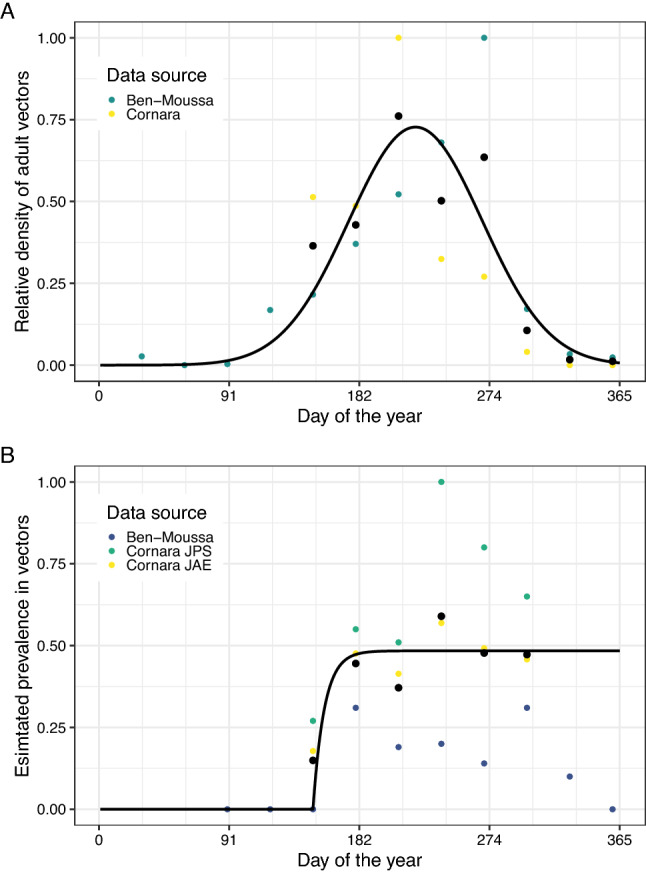
There is pronounced seasonal variability in the density of adult *P. spumarius* and the prevalence of *X. fastidiosa* infection amongst these. (**A**) Data suggest that adult *P. spumarius* are absent from January to March, and peak in density around August. This plot shows the modelled change in relative *P. spumarius* density over a year, fitted to data from two papers. The black dots show the mean density from both papers^20,28^. (**B**) Data suggest that the prevalence of *X. fastidiosa* infection in adult *P. spumarius* increases rapidly between June and July, to reach a steady peak for the rest of the year. This plot shows the modelled change in the prevalence of *X. fastidiosa* infection of *P. spumarius* over a year, fitted to data from three papers: “Cornara JPS”^28^, “Cornara JAE”^22^, and “Ben-Moussa”^20^. The black dots show the mean prevalence estimates from all three papers.

**Figure 3:**
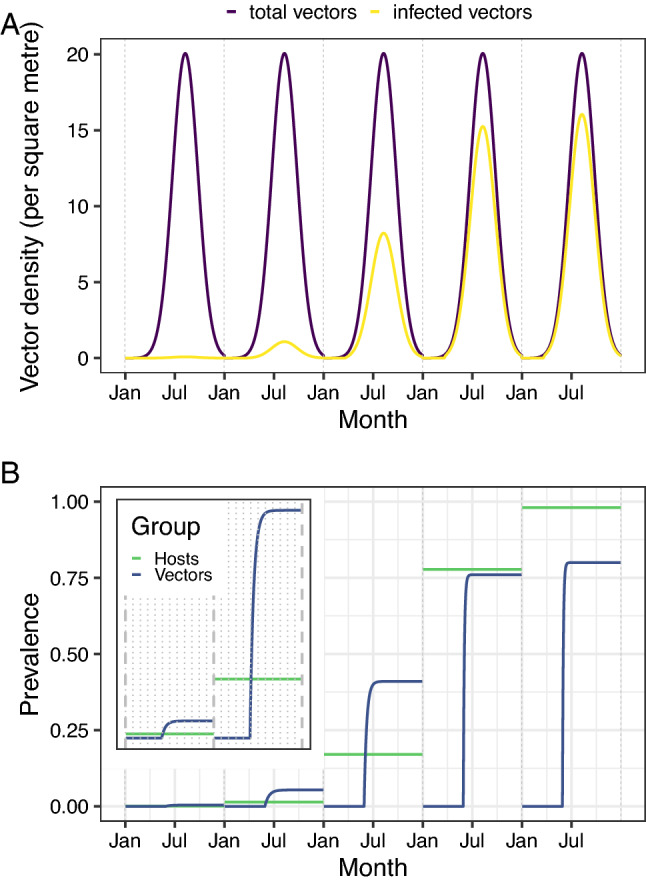
In the early stages of the epidemic, the apparent prevalence of *X. fastidiosa* in vectors increases faster than that in hosts. (**A**) Although the total density of *P. spumarius* is assumed to be fixed between years, the density of infected vectors increases each year. This plot shows the modelled density of *P. spumarius* and the density of *X. fastidiosa*-infected *P. spumarius* over the course of 5 years. (**B**) The apparent prevalence of *X. fastidiosa* infection is higher in vectors than in hosts in the early stages of a new epidemic. This plot shows how the modelled apparent prevalence of *X. fastidiosa* in hosts contrasts with that in vectors, over the course of 5 years. The inset plot shows the estimates from the first 2 years in more detail.

As a result of intensive vector surveys following the first detection of *X. fastidiosa* in Apulia, some data are available on both the abundance of adult *P. spumarius*^20,28^ and their prevalence of infection with *X. fastidiosa*^20,22,28^ over the course of a year. We captured the associated prevalence of infection in olive hosts by estimating the overall mean prevalence in these hosts over the same time period (between 2013 and 2015) and in the same area of Lecce province captured in the vector data. At the same time the peak vector prevalence was 0.48 (Fig. 2B), the prevalence amongst hosts was 0.23. Although this does not represent the very early stage spread we are predominantly interested in, we use these data to extrapolate this. We capture the temporal trends in *P. spumarius* abundance and prevalence using nonlinear regression (Fig. 2A,B). and then use an epidemiological model of spread between hosts and vectors (described in more detail in Supplementary Information B) to simulate spread between vectors (accounting for the seasonal trends in both density and prevalence) and hosts (in which the prevalence increases over consecutive years according to the total density of bacteria-carrying vector days over the course of the previous year). From this model, we are able to estimate how the prevalence of *X. fastidiosa* in both vectors and hosts would be expected to change both within and between seasons for years in which we do not have data (Fig. 3A,B). Due to the limited available data on the trends in vector prevalence over time, we also repeated these analyses using low (0.20) and high (0.70) estimates of the peak vector prevalence for the year in which we have data, as shown in Supplementary Information D.

We assess the implications of these results for surveillance by adapting our previous work on early detection surveillance in a host-vector system^13^ for a scenario in which no detections are made (as described in Supplementary Information C). This approach requires a single estimate of the ratio of apparent vector and host prevalences during early stage spread. As this ratio varies both within and between years in the case of *X. fastidiosa* (Fig. 3B), we consider only very early stage spread. If we assume that vector sampling is conducted when vector densities are at their peak (which is most logistically feasible and therefore commonly practiced in the field, as well as relating to a maximal vector prevalence in this particular case), we can estimate the initial ratio of detectable vectors and detectable hosts at this timepoint either analytically (see Supplementary Information C for more information) or directly from the model. Using this estimate, we can then estimate the maximum apparent prevalence in hosts for any given number of hosts and/or vectors inspected/tested and found to be negative, and convert this to an estimate of the true host prevalence under the assumption of logistic growth using the methods described above. As our previous work has shown that the total surveillance costs required in order to detect infection at or before a given prevalence are generally minimised when either hosts only or vectors only are sampled^13^, we only consider these two scenarios here (rather than a mixed surveillance strategy in which both hosts and vectors are sampled). Finally, we incorporate sampling and testing costs and estimate the total costs of either host or vector sampling. As the relatively low numbers of bacteria in infected individuals^21,32,33^ limit the ability to detect *X. fastidiosa* infection in vectors using ELISA tests^34^, we consider PCR testing^35^ of vectors here.

## Results

### Is visual inspection useful for detection surveys?

**Figure 4:**
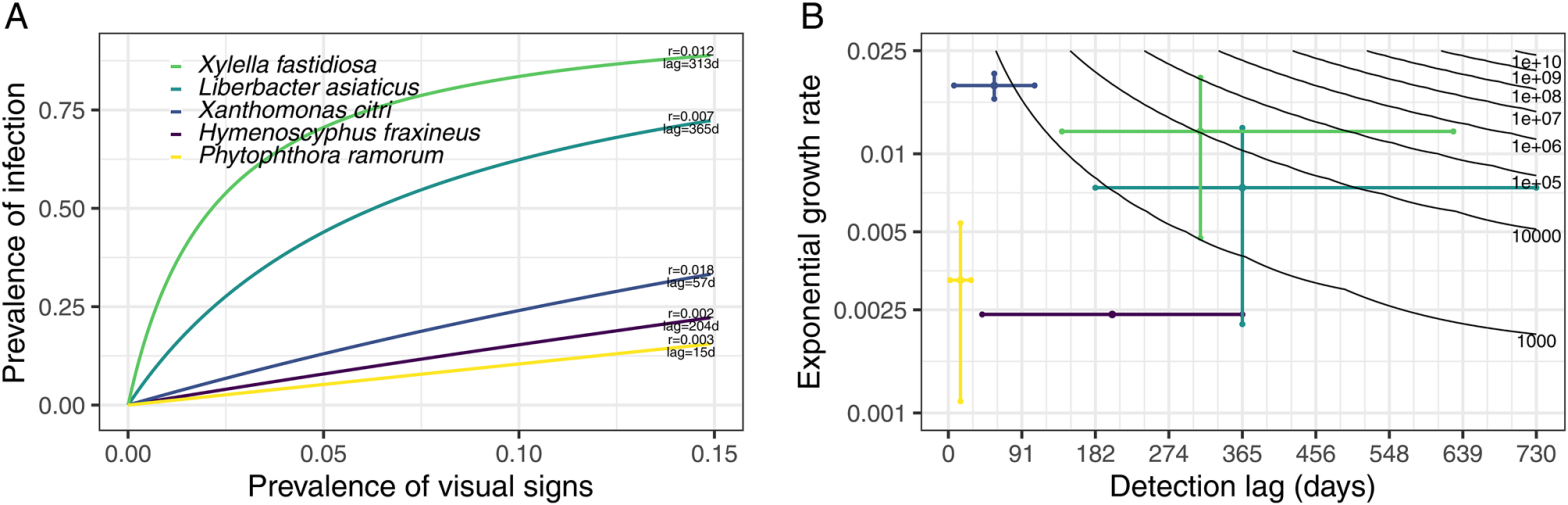
The asymptomatic period for *Xylella fastidiosa* makes it very difficult to detect at an early stage when using visual inspection. (**A**) The highest difference between apparent and true prevalence is seen for olive quick decline syndrome, caused by *X. fastidiosa*. This plot shows the relationship between the apparent prevalence (on the x-axis) and the true prevalence (on the y-axis) for a number of different pathogens (associated disease): *Hymenoscyphus fraxineus* (ash dieback); *Xanthomonas citri *subsp. *citri* (citrus canker); *Candidatus* Liberbacter asiaticus (huanglongbing); *X. fastidiosa* ST53 (olive quick decline syndrome); *Phytophthora ramorum* (ramorum). (**B**) In order to confidently declare pest freedom, more samples are needed when the asymptomatic period and/or the spread rate are high. This plot shows the relationship between the detection lag (x-axis), the exponential growth rate (on a logarithmic scale on the y-axis), and the number of samples (also on a logarithmic scale, in the contour lines) required to be 90% confident that the true prevalence is lower than 1% given that no positive detections are made. The coloured lines indicate our best estimates of the growth rate and presymptomatic period for the pathogens considered^10^.

The number of hosts which must be found to be asymptomatic to declare pest freedom is affected by the rate of pathogen spread and the duration of the presymptomatic period, which will vary for different pathogens. These factors together determine the degree of disparity between the apparent prevalence (the proportion of hosts with visual symptoms) and the true prevalence of infection. Using the mean estimates of spread rate and presymptomatic period summarised in Ref.^10^, we find that there is little difference between the apparent and true prevalences for some tree pathogens (such as *Phytophthora ramorum* or *Hymenoscyphus fraxineus*). In these cases, relatively small numbers of hosts must be inspected in order to be able to declare pathogen freedom (Fig. 4). However, using our own estimates for *X. fastidiosa* ST53 (Supplementary Table 2), we found that the disparity between the apparent and true prevalences was more marked than for any other pathogen considered, with around 80% of hosts being infected by the time 10% become detectable (Fig. 4A). The low numbers of symptomatic hosts which would be expected during early stage spread means a total of 10,384 trees would need to be observed (and all found to be asymptomatic) to be 90% confident that the prevalence of *X. fastidiosa* ST53 was lower than 1%, under our best estimates of the growth rate (0.0122 infections/infected host/day) and presymptomatic period (313 days). This is greater than for any of the other pathogens considered (Fig. 4B).

### What characteristics of a host diagnostic test would make it more cost effective than visual inspection?

**Figure 5:**
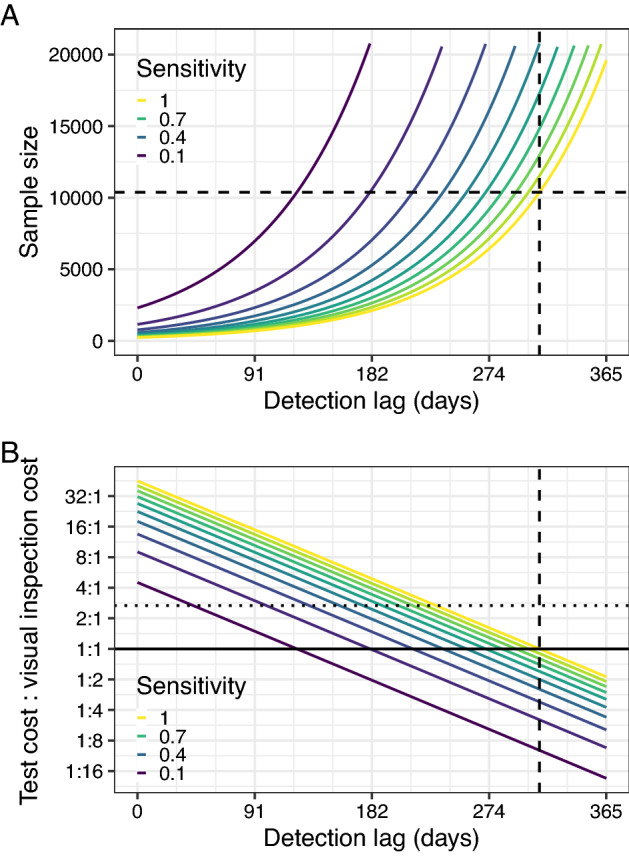
The low sensitivity of current diagnostic tests when applied to presymptomatic hosts may limit their ability to detect infections at a low prevalence. (**A**) Diagnostic tests can result in a lower sample size than visual inspection, but if the diagnostic sensitivity is low, the test needs to be able to detect infection shortly after infection. This plot shows the impact of reducing the detection lag and the diagnostic sensitivity on the number of hosts which must be found to be negative to be 90% confident that the prevalence is lower than 1% (the sample size). As the dashed lines reflect the detection lag and required sample size under visual inspection, all solid lines below the horizontal dashed line indicate that fewer trees must be tested to declare pathogen freedom than would have to be visually inspected. (**B**) Lower detection sensitivities and higher costs both reduce the feasibility of a nonvisual detection method, even if the detection lag is short. This plot expands on plot A to also incorporate testing costs. We capture this by showing in solid coloured lines the relative cost of an alternative detection method at which the total costs of surveillance would be equal to those under visual inspection, for different diagnostic sensitivities. The intersections of the solid coloured lines with the solid black line (which indicates that the costs of the detection method is equal to that of visual inspection) therefore represent equal required sample sizes (and therefore match the intersections of the curves in plot A with the dashed line in that plot). The horizontal dotted line indicates the current estimated relative cost of using the host ELISA test (a cost ratio of €14.63/€5.48 = 2.67). The vertical dashed line shows the presymptomatic period for *X. fastidiosa* (and therefore the detection lag for visual inspection). All areas of the parameter space below the test sensitivity contour of interest indicate that the alternative detection method is cheaper to deploy than visual inspection, and all areas above indicate that visual inspection is cheaper.

Although detection methods able to detect the pathogen before the development of symptoms (i.e. with shorter detection lags) require fewer samples to be collected (Fig. 5A), any associated reductions in the diagnostic sensitivity increases the required sample size. There is therefore a trade-off between the detection lag and the diagnostic sensitivity, meaning that both of these test characteristics must be considered together. Although the most marked reduction in sample size is associated with relatively small reductions from the original detection lag, there are considerable increases in required sample size when the sensitivity of detection is low (Fig. 5A). In practical terms, a test with a detection lag period half that of visual inspection would require fewer samples than visual inspection if the diagnostic sensitivity of this test was over 0.15.

However, the detection lag and diagnostic sensitivity are not the only important considerations for an alternative detection method. We also need to consider how much the new method costs to deploy, and how this compares to visual inspection^12^. Assuming that the alternative detection method is an ELISA test, which costs around 2.67 times more than visual inspection to deploy (Maria Saponari, Personal Communication; Supplementary Table 2), we find that a test with a perfect sensitivity must be able to detect infection with *X. fastidiosa* at or before 232 days post-infection to be more cost effective than visual inspection (that is, the intersection of the uppermost solid coloured line with the horizontal dotted line in Fig. 5B). If the sensitivity of the test is lower, then it must be possible to detect infection even earlier than this for the test to be more cost effective than visual inspection. Although very little information is available on the performance of the ELISA test on asymptomatically infected hosts at different times post-infection, it is likely to be very low, given the large number of leaves (the majority of which will not contain bacteria) on a tree.

Our method can also be used to assess other potential host-based detection methods. Figure 5B shows how the detection lag, diagnostic sensitivity and cost influence the required sample size together by estimating the “equivalence point” at which the total cost of either visual inspection or the alternative detection method would be equal for any combination of these three factors. This equivalence point is shown in Fig. 5B for different combinations of diagnostic sensitivity, detection lag, and relative test cost (the ratio of the costs of testing a single host with the alternative detection method and by visual inspection) as coloured lines. For any given diagnostic sensitivity (i.e. selecting a single coloured line in Fig. 5B), we find that changing the relative costs effectively shifts the previous relationship between detection lag and required sample size in a linear fashion. This means that doubling the relative costs of the molecular test reduces the maximum acceptable detection lag by 57 days, all else being equal.

### Could laboratory testing of vectors outperform visual inspection?

To evaluate how vector testing would be expected to compare to host visual inspection we need to consider not just the diagnostic considerations of detection lag, diagnostic sensitivity, and cost, but also any differences in the prevalence of infection between hosts and vectors, which will be determined by the epidemiology of the pathogen itself. We find that these epidemiological considerations are favourable for vector surveillance during early stage spread, with the prevalence of vector infection being up to four times higher than that in hosts. Although the detection lag and diagnostic sensitivity of a PCR test are also favourable for vector surveillance, the higher costs associated with such testing means that vectors must be pooled in order for these approaches to be cost-effective.

Our model of the population dynamics of adult *P. spumarius* replicates the seasonal fluctuations in adult *P. spumarius* density (Fig. 3A) and prevalence of infection with *X. fastidiosa* (Fig. 3B) seen in the data (Fig. 2). In line with the available data (Fig. 2B), the prevalence of infection is zero when adults are not present, before rising as adults emerge and initially feed on olive hosts^36,37^, and then remaining unchanged for the remainder of the year as the total density of adults declines (which we term the asymptotic prevalence). Our model is also able to predict how the prevalence of *X. fastidiosa* amongst adult *P. spumarius* varies over a number of years (Fig. 3B). Despite the similar general trend each year, the asymptotic prevalence in vectors increases over the first 4 years, as does the prevalence in hosts (Fig. 3B). However, these are not symmetrical increases—with the vector prevalence reducing from 4.06 times higher than the host prevalence in the first year to 3.86 times higher in the second year and 2.40 times higher in the third year. The fact that this ratio remains greater than 1.0 shows that during early stage spread, any given number of sampled vectors would have a higher probability of containing an infected vector than an equal number of sampled hosts.

**Figure 6:**
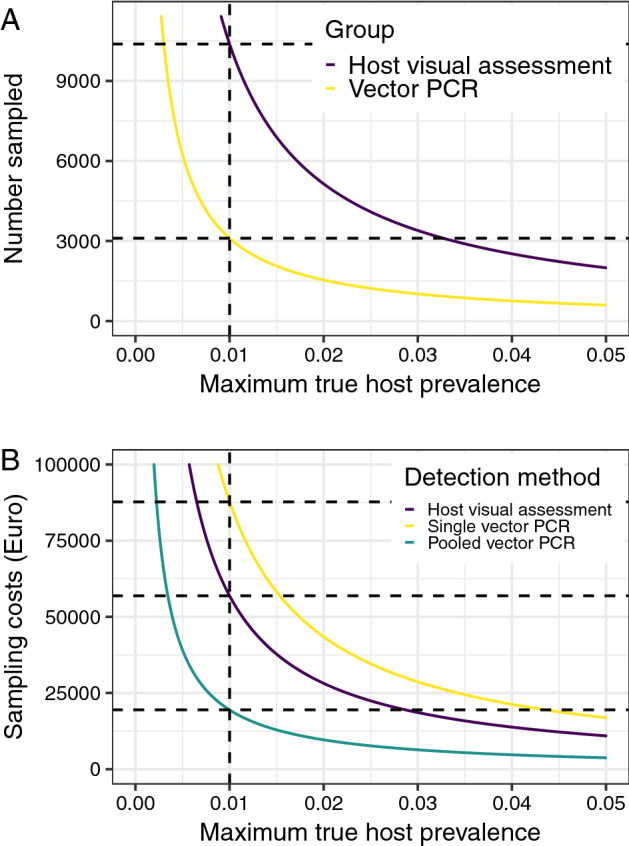
In cases where *X. fastidiosa* is not thought to be present, fewer vectors than hosts need to be tested in order to declare pest freedom. (**A**) The number of hosts which need to be tested to detect at a given prevalence is over three times higher than the number of vectors. This plot shows the 90th percentile of the host prevalence in the absence of positive detections on the x-axis, and the number of individuals which would have to be sampled (and found to be negative) to achieve this on the y-axis, when hosts or vectors are sampled exclusively. The intersection of the curves and the vertical dashed line represents the sample size required to be 90% confident that the true prevalence is lower than 1% if no detections are made. (**B**) If vectors are pooled, the total cost of sampling hosts is around three times higher than the cost of sampling vectors. This plot shows the 90th percentile of the host prevalence in the absence of positive detections on the x-axis, and the total cost of the required sampling and testing effort to achieve this on the y-axis, when hosts or vectors are sampled exclusively. We assume that hosts are sampled with visual inspection and ELISA confirmation of suspected positives, and that vectors are tested using qPCR, either singly or pooled in batches of five.

When we consider only the differences in detection lag and diagnostic sensitivity between vector and host sampling, we find that a total of 10,384 hosts would need to be sampled to be able to declare a prevalence lower than 1%, in contrast to 3106 vectors (Fig. 6A). However, when we account for the fact that laboratory testing of single vectors is higher than the costs of host visual inspection, we find that it would cost €87,754 to reliably declare pest freedom when sampling vectors in contrast to the €56,902 required for host visual inspection (Fig. 6B). Studies have suggested that vectors can be pooled in batches of up to five insects^38^. We estimate that doing this would reduce the costs to €19,444, assuming that this pooling does not impact upon the test sensitivity (Fig. 6B).

We also explored the impact of lower and higher peak vector prevalences in relation to the known host prevalence (Supplementary Information D). We found that a lower vector prevalence (more in line with one of the two available studies^20^) resulted in the prevalence of vector infection during early stage spread being approximately equal to that of hosts and therefore removing the advantages of vector surveillance. However, a higher vector prevalence (in line with the other study^28^) further heightened the value of vector surveillance, with early stage prevalences being over ten times higher in vectors than in hosts. In this scenario, even testing vectors individually resulted in lower surveillance costs than host inspection.

## Discussion

### Summary

The rate of new plant pathogen invasions has skyrocketed in recent years, associated with increases in international travel and trade and changes in land use and climate^1–3^. This is exemplified by the recent detection of the vector-borne plant pathogen *Xylella fastidiosa* in numerous European countries, reflecting a number of separate incursions^39^. Given the considerable threats this pathogen poses to plant health throughout the continent were it to spread further, as well as the continued threat of incursion of other plant pathogens, we are faced with the question of how best to conduct surveillance to ensure that “pest free areas” remain as such. These surveillance activities (known as “detection surveys”) must be capable of detecting the presence of the pathogen at low prevalences, and to date, have predominantly relied on the visual inspection of host plants for signs of disease. Whilst this remains the only plausible method of detecting new, unexpected, pathogens or disease syndromes, it is unclear whether the wealth of alternative detection strategies offered by advances in molecular diagnostics and image analysis may be more appropriate when the pathogen of interest is known. We investigate whether visual surveillance can still be justified for *X. fastidiosa* detection surveys by comparing the expected performance of visual inspection to that for other tree pathogens and then evaluating the performance of alternative host-based methods such as molecular diagnostic tests and laboratory testing of insect vectors in relation to visual inspection. Although directly valuable for informing future surveillance for *X. fastidiosa*, our results allow us to better understand the situations in which these different detection methods may be best applied, and the constraints to their use.

Although *X. fastidiosa* has over 600 known potential host species^40^, we focus here on the *X. fastidiosa* ST53 – olive system, as found in Apulia, Italy^41^. In this system, the combination of a high spread rate and a long presymptomatic period means that visual inspection is likely to fail to reliably detect invasions at an early stage of invasion (i.e. when the prevalence of infection is very low) unless very large numbers of hosts are inspected (Fig. 4). Although fewer hosts would need to be inspected if molecular tests capable of reliably detecting infection before the development of symptoms were used, it is unlikely that this reliable detection can be achieved with these tests (since the probability of selecting a sample containing the pathogen is so low). As a result of the likely low diagnostic sensitivity associated with molecular testing of presymptomatic trees, larger numbers of trees would have to be sampled (Fig. 5A). As an additional constraint, the higher financial costs of molecular testing (even when using lower cost ELISA methods) compared to visual inspection also mean that sample size reductions would need to be very substantial before the tests become more cost effective (Fig. 5B). However, there remains some promise in the use of higher throughput, whole-tree methods such as remote sensing, which may be capable of reliably detecting presymptomatic trees at a relatively low cost per tree (due to their capacity for inspecting large numbers of trees relatively quickly), which will be explored in more detail in future work. We also find that sampling insect vectors and testing them for the presence of the pathogen has the potential to outperform both visual inspection and molecular testing of hosts (Fig. 6A). As well as offering shorter detection lag periods and higher diagnostic sensitivities, we find that the prevalence in vectors during early stage spread would be expected to be higher than that in hosts—making it more likely that infected individuals would be included in any sample, which therefore reduces the required sample size. The main challenge facing vector surveillance is the considerably higher per-sample costs of PCR testing. Although pooling vectors together for testing—a commonly used approach^38^—may solve this problem (Fig. 6B), further work is required to estimate the performance of this testing approach.

### Is visual inspection useful for detection surveys?

Visual inspection may be an appropriate detection method to use in pathogen detection surveys, but this depends on the rate of pathogen spread and the length of time before symptoms develop. As Fig. 4 shows, the sample sizes required are lowest in cases where the pathogen spreads slowly and symptoms develop quickly (in Fig. 4B, the lowest sample size contour is reached roughly when the time before symptoms develop is lower than the inverse of the spread rate). This agrees with our previous work, which showed that visual inspection for *Phytophthora ramorum* in rhododendron (a slow spreading pathogen with a short presymptomatic period) is likely to be more cost effective than the use of rapid diagnostic tests^12^. However, *X. fastidiosa* ST53 in olive both spreads rapidly and takes a long time for symptoms to develop. As a result, the number of trees which must be inspected for symptoms of *X. fastidiosa* infection during detection surveys is higher than for any other tree pathogen considered here (Fig. 5B). Although this intensity of surveillance is comparable to that in recent years within the 10 km wide ‘buffer zone (‘Zona Cuscinetto’) adjacent to the known infected zone in Apulia, it likely represents an unfeasibly high surveillance effort to maintain for long periods of time over the large areas for which such surveillance would be required (such as the remainder of Apulia, or even Italy as a whole). We assume that visual detection has both a perfect diagnostic specificity (i.e. that inspectors would be able to correctly identify all uninfected hosts) and a perfect diagnostic sensitivity (i.e. that inspectors would be able to detect all infected hosts after 313 days of infection). Our assumption of a perfect diagnostic specificity (i.e. that inspectors would not mistake other conditions for *X. fastidiosa* infection in uninfected hosts) corresponds to the guidance that any suspected cases would undergo confirmatory laboratory testing^24^, thereby making false positives unlikely. Our assumption of a perfect diagnostic sensitivity is a “best case” scenario, and a lower sensitivity (for example, resulting from nonspecific or subtle symptom development) would further increase the required sample sizes (Fig. 5A). Although little is known of the true sensitivity of visual inspection for plant pathogens, the impact of variability in both symptom development and in inspector performance would be a valuable avenue for future study.

### What characteristics of a host diagnostic test would make it more cost effective than visual inspection?

A number of novel methods of detection of host infection have become available in recent years. Although our method is flexible enough to be applicable to any of these, we focus mainly on the use of molecular tests—in particular, ELISA tests—which are currently being deployed in the field alongside visual inspection. Although theoretically capable of detecting presymptomatic infection in hosts and being relatively cheap to deploy, less is known of the diagnostic sensitivity of these tests in the field. Although estimates of test sensitivity are available, these are generally based upon the testing of either symptomatic or known infected tissue, and thus do not account for the fact that the pathogen is not homogeneously distributed throughout infected hosts—particularly early in infection. This is a particular issue for tree pathogens, and therefore means that there is a high probability that tissue sampled from an infected host will either not contain the pathogen at all or only at low levels. Given that even cheaper ELISA tests cost over two and a half times that of visual inspection, the number of hosts which need to undergo testing must be less than 40% of the number of hosts requiring visual inspection for the total surveillance costs of both methods to be equal. This can be achieved with a molecular test able to detect infection 6 months after infection (i.e. around 5 months before symptoms develop) and a host-level diagnostic sensitivity of greater than 0.7. To the authors’ knowledge, no available molecular tests could be expected to have sensitivities this high at this stage of infection. Although advances in molecular diagnostics offer some potential for reducing the costs of diagnostic tests, until a method of accurately selecting infected tissue for testing is developed, we therefore conclude that molecular testing of host plants is likely to remain of limited use for detection surveys.

Amongst nonvisual detection methods, non-molecular methods such as aerial remote sensing^42^ or canine olfactory detection^43,44^ may be capable of more reliably identifying infected hosts before the development of symptoms as they operate at the level of the whole tree rather than a particular sample, and may therefore be more appropriate for detection surveys than either visual inspection or molecular testing. Another potential advantage of these particular methods is that they are potentially capable of screening large numbers of hosts in a short space of time, meaning that their cost of deployment on an individual host basis could be relatively low.

### Could laboratory testing of vectors outperform visual inspection?

We finally consider whether surveillance of insect vectors could circumvent some of the challenges associated with host surveillance. The concept of testing vectors for pathogens is a recognised component of surveillance for emerging vector-borne pathogens of humans and other animals^45,46^, as well as of plants^24^. Indeed, the first detection of the citrus pathogen *Candidatus* Liberibacter asiaticus (the cause of the citrus disease huanglongbing) in California was made in insect vectors^47^. However, to date, most vector surveillance for *X. fastidiosa* has focused on the identification of competent vectors, seasonality of infection, and the spatial limits of the pathogen^48–50^. We find that the high prevalences of vector infection during early stage spread, the potential for reliable detection early in infection, and the ability to reduce testing costs through pooling, all make insect vectors a potentially valuable “sentinel host” for the detection of *X. fastidiosa* ST53 at low prevalences of host infection.

Although the short latent period and the reliable localisation of the pathogen in infected vectors suggests that vector surveillance could reduce the long detection lags and low diagnostic sensitivities which constrain host surveillance, very little data are available on how the prevalence in vectors relates to that in hosts during early stage spread. We therefore estimate this using a mechanistic model, created to reflect the population dynamics and infection of the main vector of *X. fastidiosa*, *P. spumarius*, in Apulia. Although our precise findings are therefore specific to the Apulian scenario, our framework is generic and can be adapted to other settings or other pathosystems if desired. From our model we obtain a rule of thumb which determines the relative prevalence in vectors compared to that in hosts, and therefore tells us something of the relative value of conducting surveillance in vectors. This ratio increases as the host density or the rate of pathogen acquisition by vectors increases and decreases as the rate of vector emergence increases. The inverse relationship between the vector and host densities and the relative prevalences in each initially appears counterintuitive, but represents the potential total increase in inoculum and therefore the infection pressure from vector to host or from host to vector, respectively.

Using our best estimates of these parameters in the Apulian scenario, our model predicts that the apparent prevalence of *X. fastidiosa* infection in these vectors would be around four times higher than that in hosts in the early stages of infection. This means that lower sampling rates would be required in vectors than in hosts during early pathogen spread to reliably sample infected individuals. This high vector prevalence is supported by the observed rapid spread of *X. fastidiosa* ST53 between Apulian olive trees by *P. spumarius* despite the limited transmission window each year (when adults are present and feeding on olive). However, we found that these conclusions are sensitive to the estimated relative prevalences in vectors and hosts, with lower vector prevalences removing this value of vector surveillance and higher vector prevalences heightening it (Supplementary Fig. D). As a result, further data on both host and vector infection during early stage spread for future pathogen incursions are urgently needed to verify these conclusions, especially given that studies in Spain have suggested that that there may be variability in the vector prevalence year on year^51^. We also note that there is some evidence that the prevalence amongst vectors declines towards the end of the year in Apulia^20^ (and in other locations^51^). We do not explicitly capture this decline in our model because the low numbers of vectors at this point (Fig. 3A) would be expected to have relatively little impact on transmission, and because this decline does not affect our detection ability since we assume that vectors are sampled earlier in the year when they are at high densities.

Although we assume that the diagnostic sensitivity of vector PCR is reasonably high (resulting from recent advances in vector PCR testing diagnostic methods and protocols^49,52^), PCR tests are expensive and laborious to undertake. As a result, we found that although fewer vectors need to be sampled than hosts, the costs of testing these individually was higher than that of hosts. However, assuming that the performance of PCR is unaffected when five insects are pooled together (as has been suggested^38^), the total costs of vector testing are lower than those of visual inspection (Fig. 6B). Given that the costs and performance of these molecular tests are likely to improve over time in line with advances in molecular diagnostics, these results suggest that vector testing also offers great future potential for improving the early detection of *X. fastidiosa*.

As mentioned above, our current results consider the scenario in Apulia, Italy, where *X. fastidiosa* is thought to have spread rapidly since first introduction^53^. When considering surveillance activities in other locations, the impact of factors such as climate, host availability and vector density on pathogen spread and on the value of vector surveillance will need to be explored. Finally, whilst our method can quantify the value of vector surveillance from a scientific perspective, the most appropriate response to a positive detection is best considered by decision makers. Further work will be needed to develop appropriate responses to detection in vectors, given that it is not possible to perform repeat confirmatory tests (as is possible with host trees) and that less information can be gained on the spatial distribution of infection. As a result, vector surveys alone are currently not considered sufficient to determine the *X. fastidiosa* status of any area in the European Union^25^.

## Conclusions

The rapid rate of spread of *X. fastidiosa* ST53 in olive and the considerable delay between infection and the development of symptoms makes visual inspection less able to identify low prevalences of infection required for effective detection surveys. Whilst molecular tests can reduce the delay before infection can be detected, the relatively low diagnostic sensitivity and high costs of these tests mean that they are unlikely to outperform visual inspection in the field. However, the combination of a short interval between infection and reliable detection and the high initial prevalences of infection amongst the insect vectors responsible for pathogen spread means that vector sampling offers great potential for a sustainable and effective surveillance strategy. Whilst individual testing of vectors is unlikely to currently be a cost-effective alternative to visual inspection, costs can be substantially reduced when insects are pooled together for testing.

## Supplementary Information

Supplementary Information 1.

Supplementary Information 2.

Supplementary Information 3.

Supplementary Information 4.

Supplementary Information 5.

Supplementary Information 6.

Supplementary Information 7.

Supplementary Information 8.

Supplementary Information 9.

Supplementary Information 10.

Supplementary Information 11.

Supplementary Information 12.

Supplementary Information 13.

## Data Availability

All code required to generate and analyse the data in this study is included in the Supplementary Information.
