# Supplementary material for: Epidemiologically-based strategies for the detection of emerging plant pathogens
Source: Sci Rep. 2022 Jun 29;12:10972. doi: 10.1038/s41598-022-13553-y (PMC9243127; doi:10.1038/s41598-022-13553-y)
Supplement: Supplementary file 6 — Supplementary Information 6. [file 41598_2022_13553_MOESM6_ESM.docx]

**Epidemiologically-based strategies for the detection of emerging plant pathogens.**

**Supplementary Information D: Sensitivity analysis.**

**Reduced vector prevalence**


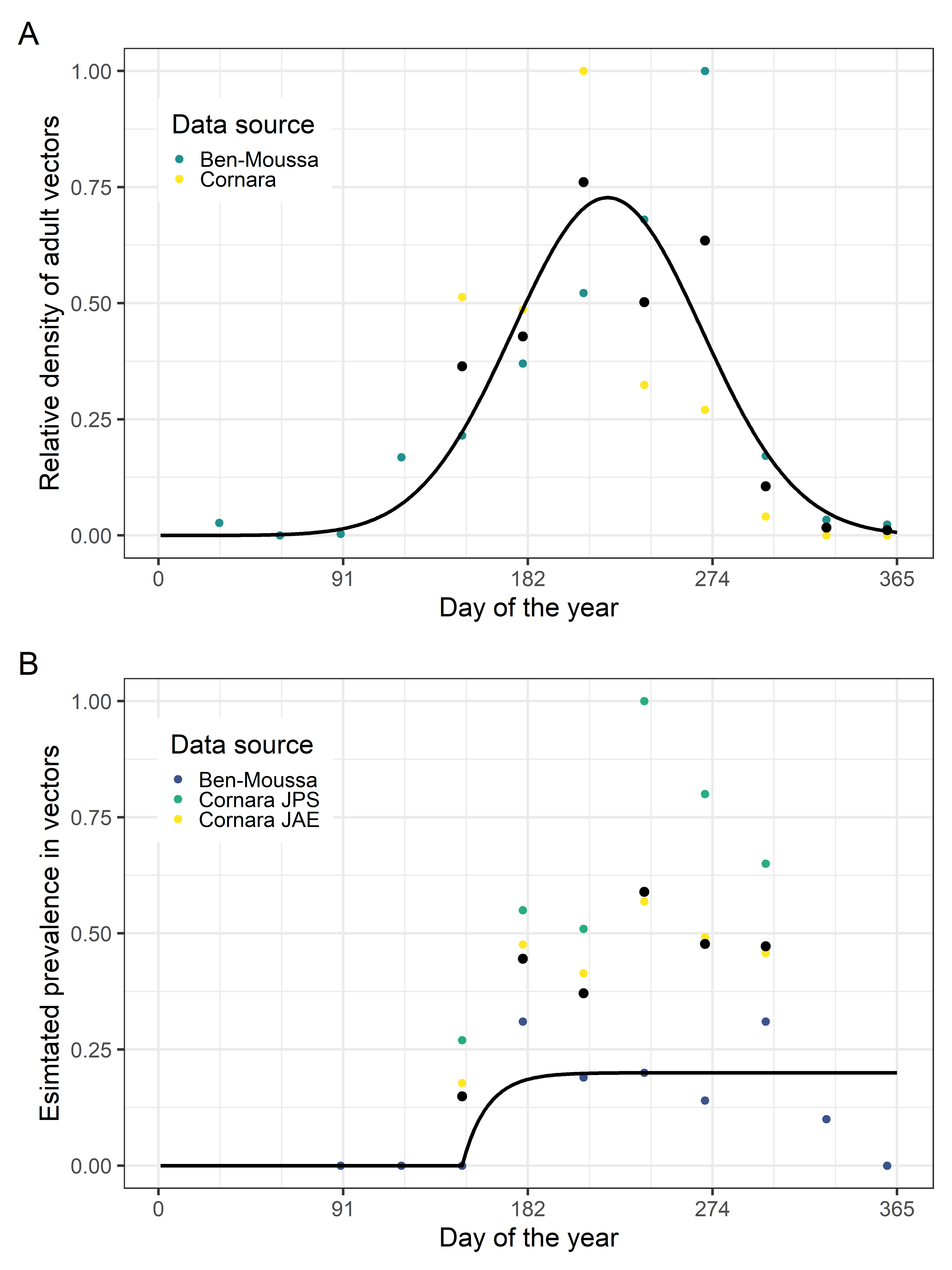


**Supplementary Figure D 1. Estimated vector densities and prevalences under the low vector prevalence parameterisation.**

A: The fit to the density data is unchanged from the original analysis. Black dots show the mean density from both papers, as described in the main text.

B: Under the new parameterisation, we obtain a reasonable fit to the Ben Moussa data, which gave a much lower plateau prevalence. Black dots show the mean prevalence estimates from all three papers, as described in the main text.


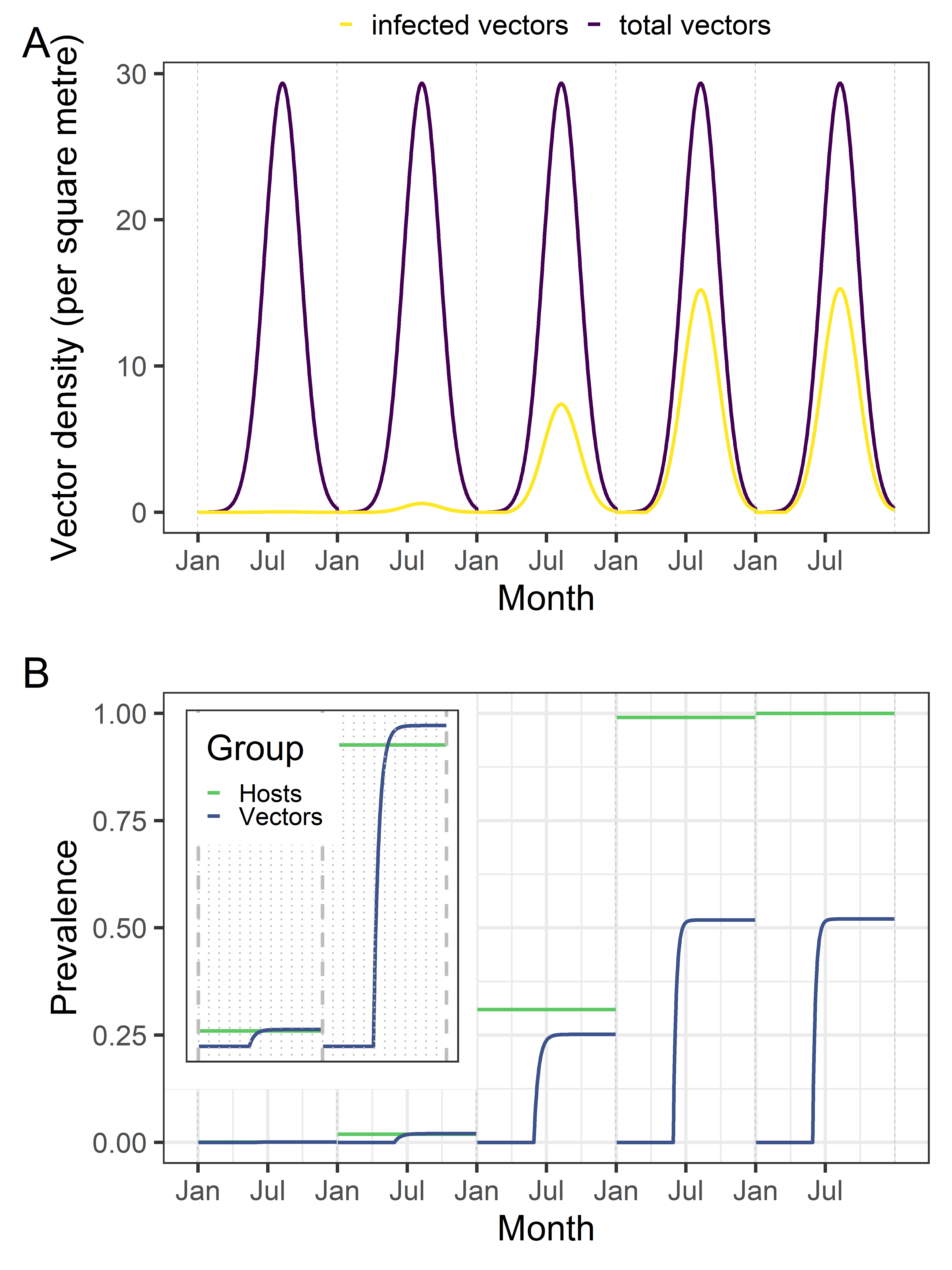


**Supplementary Figure D 2. When the prevalence in vectors is reduced, the ratio of vector and host prevalences during early stage spread is also reduced.**

A: This plot shows the modelled density of P. spumarius and the density of X. fastidiosa-infected P. spumarius over the course of five years.

B: Under this parameterisation, the prevalence of X. fastidiosa infection is slightly higher in vectors than in hosts in the early stages of a new epidemic. The inset plot shows the estimates from the first two years in more detail. The model-derived ratio of vector and host prevalences in each year is 1.09, 1.06, 0. 81, 0.52, and 0.52.


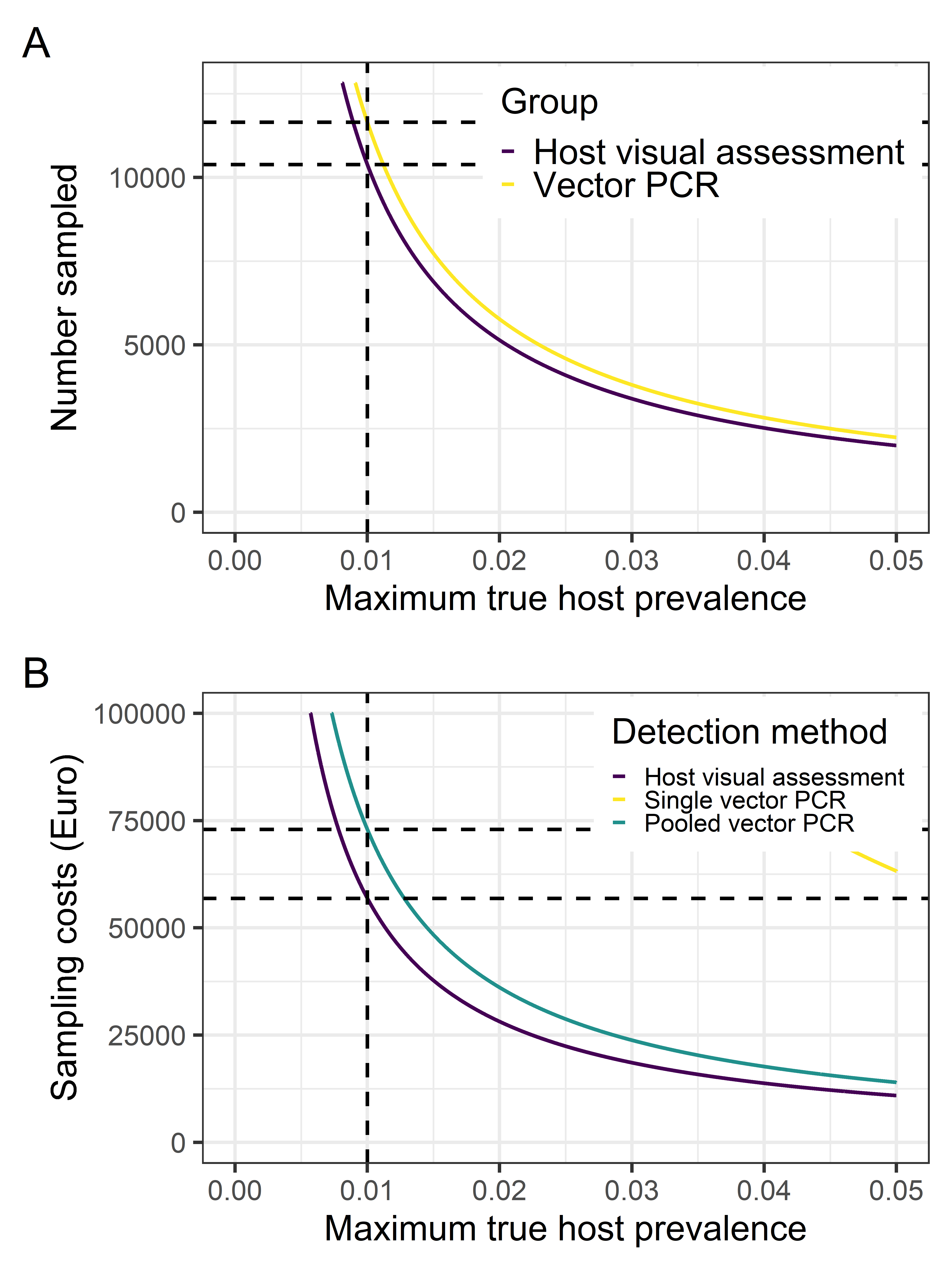


**Supplementary Figure D 3. Under the low vector prevalence parameterisation, vector testing no longer outperforms visual inspection, even in the presence of pooling.**

A: Under the new parameterisation, the number of hosts which would need to be inspected visually and found to be negative to declare at maximum host prevalence of 1% is 10,384, whereas 11,650 vectors would need to be tested and found to be negative.

B: Under the new parameterisation, the total cost of host visual inspection is €56,902 and pooled vector testing is €72,950.

**Increased vector prevalence**


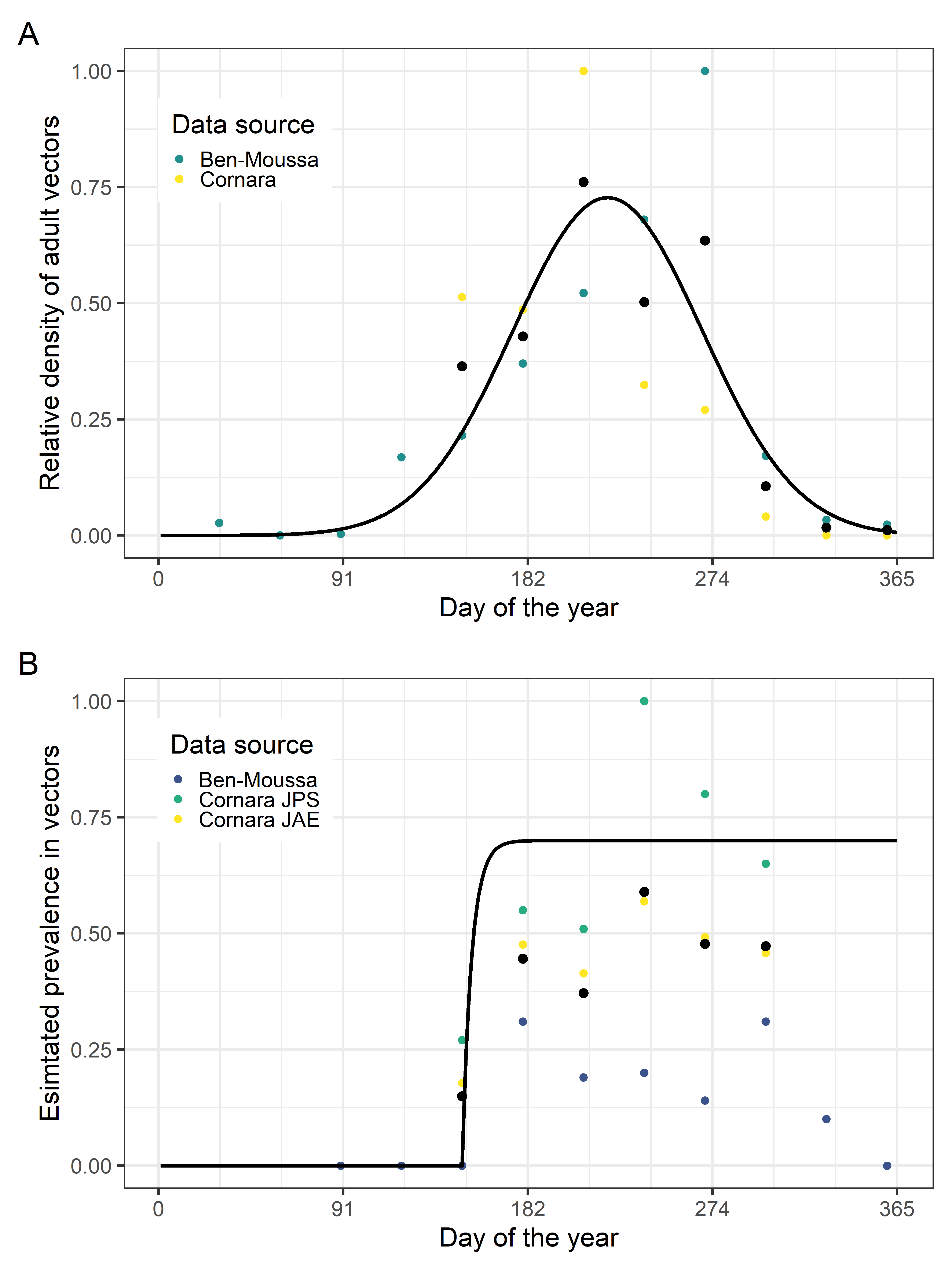


**Supplementary Figure D 4. Estimated vector densities and prevalences under the high vector prevalence parameterisation.**

A: The fit to the density data is unchanged from the original analysis. Black dots show the mean density from both papers, as described in the main text.

B: Under the new parameterisation, we obtain a reasonable fit to the Ben Moussa data, which gave a much lower plateau prevalence. Black dots show the mean prevalence estimates from all three papers, as described in the main text.


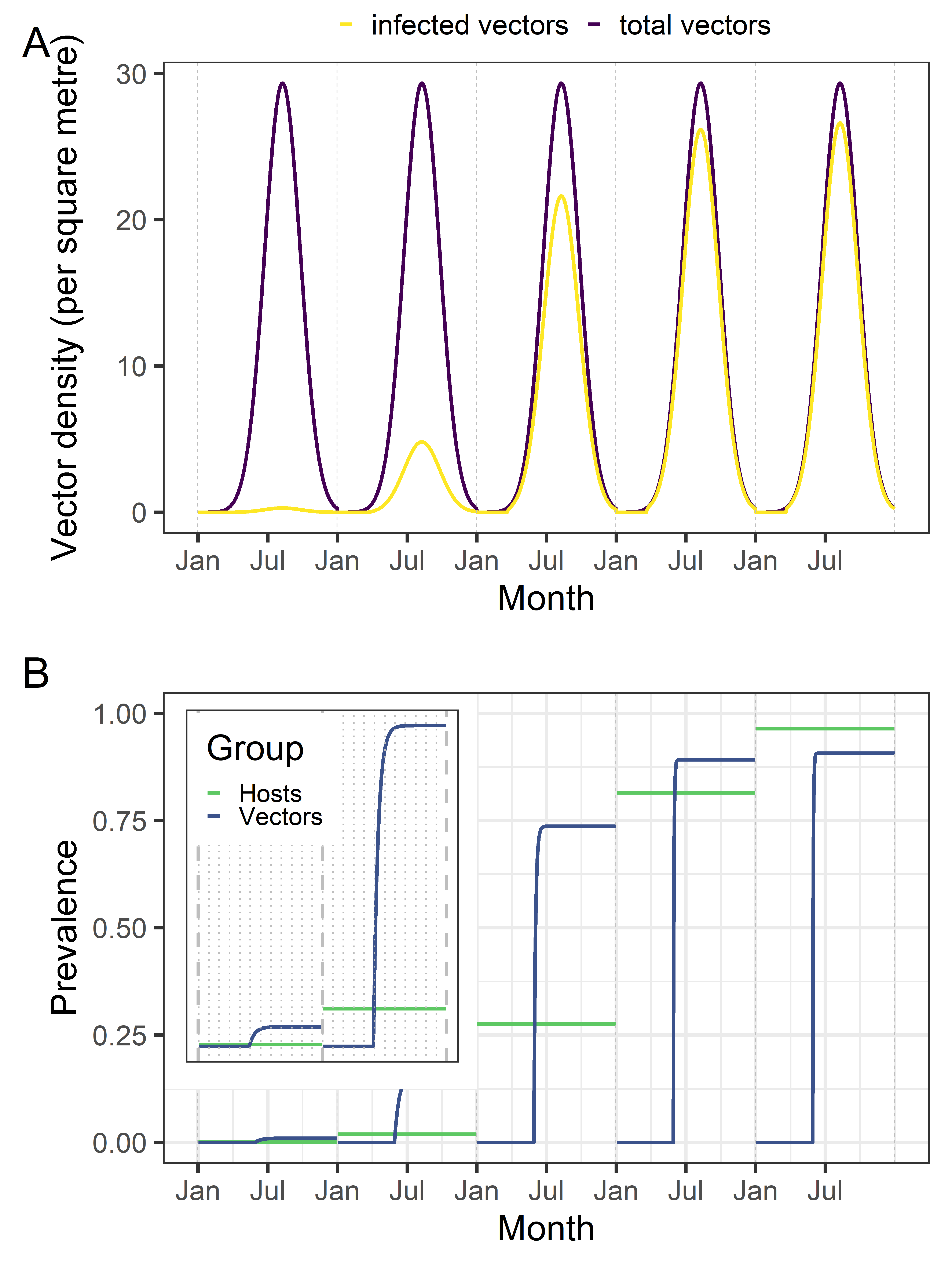


**Supplementary Figure D 5. When the prevalence in vectors is increased, the ratio of vector and host prevalences during early stage spread is also increased.**

A: This plot shows the modelled density of P. spumarius and the density of X. fastidiosa-infected P. spumarius over the course of five years.

B: Under this parameterisation, the prevalence of X. fastidiosa infection is slightly higher in vectors than in hosts in the early stages of a new epidemic. The inset plot shows the estimates from the first two years in more detail. The model-derived ratio of vector and host prevalences in each year is 10.04, 8.48, 2.67, 1.09, and 0.94.


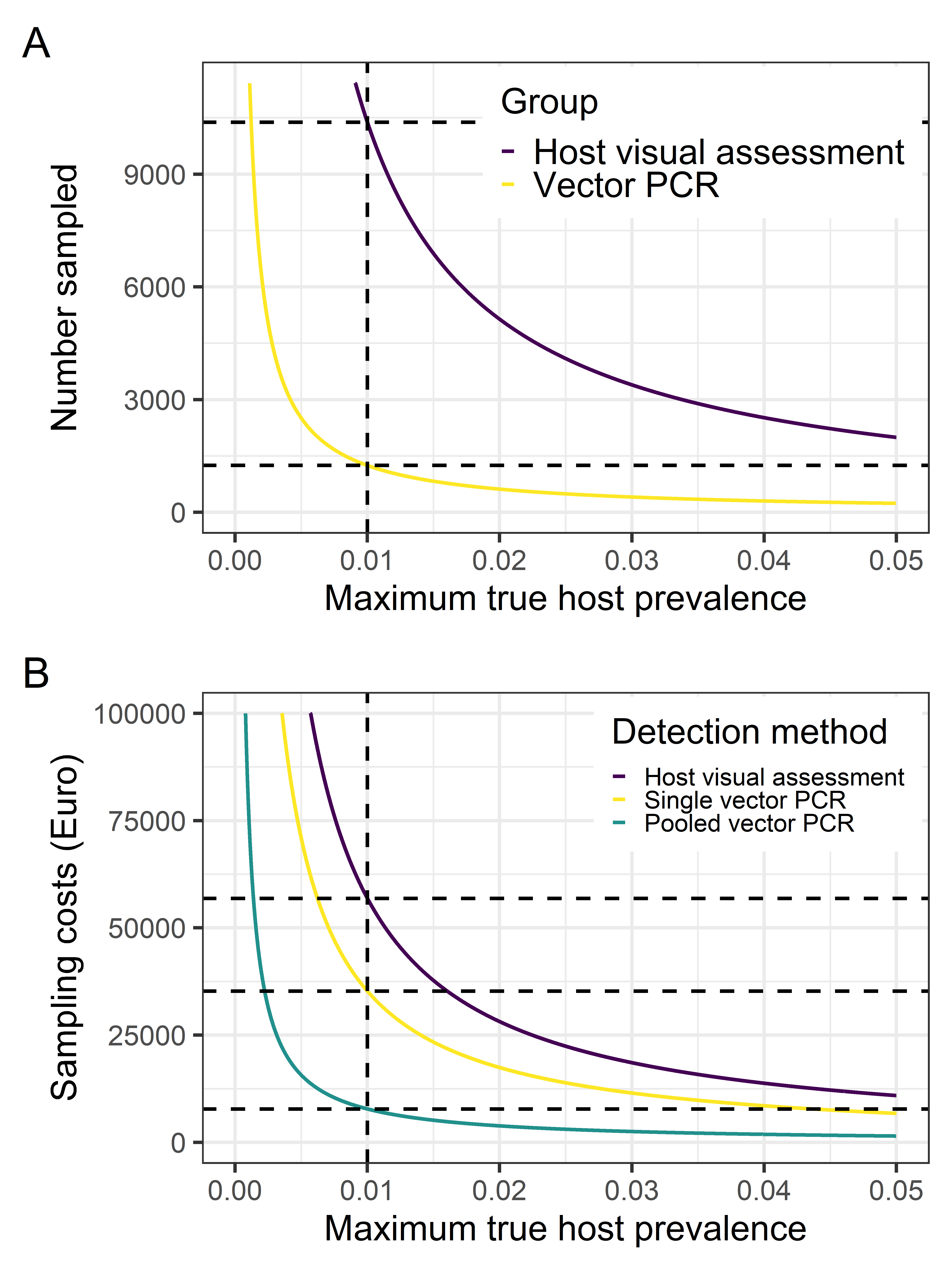


**Supplementary Figure D 6. Under the high vector prevalence parameterisation, vector testing outperforms visual inspection, even without pooling.**

A: Under the new parameterisation, the number of hosts which would need to be inspected visually and found to be negative to declare at maximum host prevalence of 1% is 10,384, whereas 1,249 vectors would need to be tested and found to be negative.

B: Under the new parameterisation, the total cost of host visual inspection is €56,902; single vector testing is €35,277, and pooled vector testing is €7,817.
