# Supplementary material for: Epidemiologically-based strategies for the detection of emerging plant pathogens
Source: Sci Rep. 2022 Jun 29;12:10972. doi: 10.1038/s41598-022-13553-y (PMC9243127; doi:10.1038/s41598-022-13553-y)
Supplement: Supplementary file 7 — Supplementary Information 7. [file 41598_2022_13553_MOESM7_ESM.docx]

**Epidemiologically-based strategies for the detection of emerging plant pathogens.**

**Supplementary Tables**

Supplementary Table 1. Surveillance parameters

| **Parameter** | **Description** | **Value** | **Units** | **Source** |
| --- | --- | --- | --- | --- |
| Maximum acceptable host prevalence |  | 0.01 | None | [1] |
| Confidence level | 1 - probability that maximum acceptable host prevalence is exceeded | 0.90 | None | [1] |

Supplementary Table 2. Host parameters

| **Parameter** | **Description** | **Value** | **Units** | **Source** |
| --- | --- | --- | --- | --- |
| Epidemic growth rate | Initial rate of exponential growth in the prevalence of infection in host | 0.0122 | New infections per infection per day |  |
| Maximum acceptable host prevalence |  | 0.001 | None |  |
| Confidence level | 1 - probability that maximum acceptable host prevalence is exceeded | 0 | None |  |
| Presymptomatic period |  | 313 | Days |  |
| Host laboratory test detection lag |  | [varied] | Days |  |
| Sensitivity of visual detection | Probability of correctly identifying an infected host visually after the asymptomatic period | 1.0 | None |  |
| Sensitivity of host laboratory diagnostic | Probability of correctly identifying an infected host using a laboratory test after the detection lag period | [varied] | None |  |
| Cost of visual inspection with no testing |  | 2.13 | Euros per tree | Assuming that a team of two inspectors (each paid €80 per day) can inspect around 75 trees per day. Estimates from expert opinion. |
| Cost of host laboratory diagnostic |  | 12.50 | Euros per test | From expert opinion (Maria Saponari). |
| Total cost of host laboratory testing |  | 14.63 | Euros per test | The sum of the cost of visiting a tree and the cost of a laboratory diagnostic |
| Total cost of visual inspection surveillance |  | 5.48 | Euros per tree | Based on estimates of proportions of (uninfected) trees which underwent ELISA testing in 2017. |

Supplementary Table 3. Host-vector model parameters

| **Parameter** | **Description** | **Value** | **Units** | **Source** |
| --- | --- | --- | --- | --- |
| Prevalence in vectors | Prevalence of infection in hosts and vectors early in epidemic, measured at the same time | 0.48 | None | [2]–[4] |
| Prevalence in hosts |  | 0.23 | None | Surveillance data, restricted to study areas in [2]–[4] |
| Acquisition rate $\left( \beta\right)$ | Rate of vector acquisition of *X. fastidiosa* from infected hosts | 21.4428 | New vector acquisitions per vector per host per day | Fitted to data [2], [4], and an estimate of symptomatic host density. |
| Inoculation rate $\left( \alpha\right)$ | Rate of host infection with *X. fastidiosa* from infected vectors | 8.579169e-06 | New host inoculations per vector per host per day |  |
| Host density $\left( D \right)$ | Density of hosts in an orchard/grove | 1/81 | Hosts per m^2^ |  |
| Maximum vector density | Peak vector density | 20 | Vectors per m^2^ | [5], [6] |
| Initial relative density at time of adult emergence | Initial density of vectors | 0.005343 | No units |  |
| Rate of emergence of adult vectors at time of adult emergence $\left( g_{0} \right)$ |  | 0.064913 | No units | Fitted to data [2], [4] |
| Rate of mortality of adult vectors $\left( a \right)$ |  | 0.0004574 |  | Fitted to data [2], [4] |
| Time of adult emergence $\left( t_{em} \right)$ |  | 80 | Julian day |  |
| Time of first adult infection $\left( t_{in} \right)$ |  | 150 | Julian day |  |
| Vector laboratory test detection lag |  | 0 | Days |  |
| Sensitivity of vector diagnostic | Probability of correctly identifying an infected vector using a laboratory test after the detection lag period | 0.82 | No units | Taken from data on vector PCR testing [7], [8] |
| Cost of collecting vectors | Cost of collecting a single vector | 0.76 | Euros per vector | Assuming that an inspector (paid €80 per day) can collect around 105 insects per day. Estimates from expert opinion. |
| Cost of testing vectors | Cost of the qPCR test for vector infection | 27.50 | Euros per test | Estimated cost of qPCR test. Estimates from expert opinion. |
| Vector pool size | Number of vectors pooled per test | 5 | Vectors | Estimates of maximum pool size for qPCR from expert opinion. |

Supplementary Table 4. Relative vector density over the course of a year

| Julian date | Relative density from Ben Moussa study[2] | Relative density from Cornara study[3] |
| --- | --- | --- |
| 30 | 0.026936 | NA |
| 60 | 0 | NA |
| 90 | 0.00337 | NA |
| 120 | 0.16835 | NA |
| 150 | 0.215488 | 0.513514 |
| 180 | 0.37037 | 0.486486 |
| 210 | 0.521886 | 1 |
| 240 | 0.680135 | 0.324324 |
| 270 | 1 | 0.27027 |
| 300 | 0.171717 | 0.040541 |
| 330 | 0.03367 | 0 |
| 360 | 0.023569 | 0 |

Supplementary Table 5. Prevalence of vector infection with X. fastidiosa over the course of a year

| Julian date | Prevalence from Ben-Moussa study[2] | Prevalence from Cornara study[3] | Prevalence from Cornara study[4] |
| --- | --- | --- | --- |
| 30 | NA | NA | NA |
| 60 | NA | NA | NA |
| 90 | 0 | NA | NA |
| 120 | 0 | NA | NA |
| 150 | 0 | 0.27 | 0.178 |
| 180 | 0.31 | 0.55 | 0.476 |
| 210 | 0.19 | 0.51 | 0.414 |
| 240 | 0.20 | 1.00 | 0.569 |
| 270 | 0.14 | 0.80 | 0.492 |
| 300 | 0.31 | 0.65 | 0.458 |
| 330 | 0.10 | NA | NA |
| 360 | 0 | NA | NA |

**References**

[1] European Commission, Commission Implementing Regulation (EU) 2020/1201 of 14 August 2020 as regards measures to prevent the introduction into and the spread within the Union of Xylella fastidiosa (Wells et al.). 2021. Accessed: Feb. 25, 2021. [Online]. Available: https://eur-lex.europa.eu/legal-content/EN/TXT/PDF/?uri=CELEX:32020R1201&from=EN

[2] I. E. Ben Moussa et al., ‘Seasonal fluctuations of sap-feeding insect species infected by Xylella fastidiosa in Apulian olive groves of southern Italy’, J. Econ. Entomol., vol. 109, no. 4, Art. no. 4, Aug. 2016, doi: 10.1093/jee/tow123.

[3] D. Cornara et al., ‘Spittlebugs as vectors of Xylella fastidiosa in olive orchards in Italy’, J Pest Sci, vol. 90, pp. 521–530, 2017, doi: 10.1007/s10340-016-0793-0.

[4] D. Cornara et al., ‘Transmission of Xylella fastidiosa by naturally infected Philaenus spumarius (Hemiptera, Aphrophoridae) to different host plants’, J. Appl. Entomol., vol. 141, no. 1–2, Art. no. 1–2, Feb. 2017, doi: 10.1111/jen.12365.

[5] F. Di Serio et al., ‘Collection of data and information on biology and control of vectors of Xylella fastidiosa’, EFSA, EFSA supporting publication 2019:EN-1628, 2019. doi: 10.2903/sp.efsa.2019.EN-1628.

[6] EFSA Panel on Plant Health et al., ‘Update of the Scientific Opinion on the risks to plant health posed by Xylella fastidiosa in the EU territory’, EFSA Journal, vol. 17, no. 5, Art. no. 5, May 2019, doi: 10.2903/j.efsa.2019.5665.

[7] F. Poliakoff, ‘Novel and high-throughput diagnostic procedures to detect Xylella fastidiosa in planta and vectors developed within POnTE project’, presented at the Second European Conference on Xylella fastidiosa, Ajaccio, Corsica, France, 2019.

[8] M. Saponari et al., ‘Harmonized protocol for monitoring and detection of Xylella fastidiosa in its host plants and its vectors (PROMODE)’, May 2019. doi: 10.5281/zenodo.2656679.
